# Transcriptomic analysis of *EGFR* co-expression and activation in glioblastoma reveals associations with its ligands

**DOI:** 10.1093/noajnl/vdae229

**Published:** 2024-12-28

**Authors:** Santoesha A Ghisai, Nastaran Barin, Levi van Hijfte, Kim Verhagen, Maurice de Wit, Martin J van den Bent, Youri Hoogstrate, Pim J French

**Affiliations:** Department of Neurology, Erasmus Medical Center Cancer Institute, Rotterdam, The Netherlands; Department of Precision and Microsystems Engineering, Delft University of Technology, Delft, The Netherlands; Department of Neurology, Erasmus Medical Center Cancer Institute, Rotterdam, The Netherlands; Department of Tumor Immunology, Erasmus Medical Center Cancer Institute, Rotterdam, The Netherlands; Department of Neurology, Erasmus Medical Center Cancer Institute, Rotterdam, The Netherlands; Department of Neurology, Erasmus Medical Center Cancer Institute, Rotterdam, The Netherlands; Department of Neurology, Erasmus Medical Center Cancer Institute, Rotterdam, The Netherlands; Department of Neurology, Erasmus Medical Center Cancer Institute, Rotterdam, The Netherlands; Department of Neurology, Erasmus Medical Center Cancer Institute, Rotterdam, The Netherlands; Department of Neurology, Erasmus Medical Center Cancer Institute, Rotterdam, The Netherlands

**Keywords:** EGFR, glioblastoma, ligands, RNA, transcriptomics

## Abstract

**Background:**

Approximately half of the isocitrate dehydrogenase (IDH)-wildtype glioblastomas (GBMs) exhibit *EGFR* amplification. Additionally, genomic changes that occur in the extracellular domain of *EGFR* can lead to ligand-hypersensitivity (R108K/A289V/G598V) or ligand-independence (*EGFRvIII*). Unlike in lung adenocarcinoma (LUAD), clinical trials with epidermal growth factor receptor (EGFR) inhibitors showed no survival benefit for GBM and it remains unclear why. We aimed to elucidate differences in molecular mechanisms of *EGFR* activation and regulation between GBM and LUAD.

**Methods:**

We used RNA-sequencing (RNA-seq) data to find *EGFR* co-regulated genes and pathways in GBM and compare *EGFR* signaling patterns between GBM and LUAD. Cellular origins of expression signals were determined by analyzing single-cell RNA-seq data.

**Results:**

We identified 2 ligands (*BTC*/*EREG*) among the significant EGFR predictor genes (TCGA-GBM: *n* = 169, Intellance-2: *n* = 166). Their expression was inversely correlated with *EGFR* amplification and incidence of ligand-sensitive mutations. Ligands were expressed by nonmalignant cells and differed in their primary source of expression (*BTC*: neurons, *EREG*: myeloid). High expression of *MDM2* and *CDK4* was less common in EGFR-amplified GBMs with ligand-sensitive mutations compared with those without these mutations. Our analyses revealed distinct transcriptional profiles between GBM and LUAD when comparing tumors carrying activating mutations.

**Conclusions:**

*BTC* and *EREG* are negatively associated with *EGFR* expression in GBM. These findings emphasize the role of ligands in regulating *EGFR*, where *EGFR* activation seems to be modulated by the highly varying levels of *EGFR* amplification, the sensitivity of the receptor toward ligands, and ligand expression levels. Ligand expression levels and *EGFR* mutations could refine patient stratification for EGFR-targeted therapies in GBM.

**Key Points**

- Ligands *BTC* and *EREG* show negative associations with *EGFR* in GBM.
- High *MDM2*/*CDK4* were less common in EGFR-amplified GBMs with a ligand-sensitive mutation.
- EGFR-activating mutations in GBM and LUAD show distinct transcriptomic profiles.

**Importance of the Study**

Glioblastoma (GBM) is the most malignant type of primary brain tumor. The high incidence of epidermal growth factor receptor (EGFR) alterations in these tumors, combined with its role as driver mutation, make EGFR an appealing target for treatment. Unfortunately, inhibition of EGFR signaling in GBM has not proven to be beneficial so far, which contrasts the strong clinical responses observed in *EGFR*-mutated lung adenocarcinoma (LUAD) patients. Therefore, we aimed to elucidate different mechanisms of activating *EGFR* signaling by examining co-expressed genes in relation to tumor type and type of mutation. We find little overlap in the genes co-expressed with *EGFR*-activating mutations between GBM and LUAD, suggesting distinct activation pathways. However, in GBM, the expression of several ligands is inversely correlated with the level of *EGFR* amplification, highlighting the importance, and potential targetability, of ligand-induced activation of EGFR.

Glioblastoma, isocitrate dehydrogenase (IDH)-wildtype (GBM) is the most malignant type of primary brain tumor and in spite of extensive chemo-radiotherapy treatment schemes and surgical resection, the median survival remains limited to approximately 8 months.^1^ Understanding the molecular profile of GBM might guide treatment options by revealing putative targets and so improve patient survival. The epidermal growth factor receptor (*EGFR*) is the most frequently altered oncogene in GBM, amplified in around half of all tumors.^2^ High-copy number amplification of EGFR is common in GBM and often takes the form of extrachromosomal circular DNA (eccDNA) fragments.^3^*EGFR* (hyper)amplification can be followed by additional genomic molecular abnormalities in the gene, mainly in its extracellular domain (ECD). These include in-frame deletion of exons 2-7 (*EGFRvIII*), resulting in a ligand-independent receptor with constitutive activity. Additionally, there are activating missense mutations in the ECD (A289, G598, and R108),^4^ which make the receptor hypersensitive to (low-affinity) ligands.^5,6^

The high incidence of *EGFR* alterations in GBM, in combination with the presumed oncogenic driver role of the protein, make EGFR an appealing target for inhibition. Indeed, the patient benefit of treatment with EGFR inhibitors in *EGFR*-mutated lung adenocarcinoma (LUAD) is well established.^7^ Unfortunately, attempts to inhibit *EGFR* signaling in GBM have not yet proven to be beneficial.^8,9^

It is interesting to note that there are differences in the types of genomic *EGFR* alterations between GBM and LUAD. As mentioned, in GBM, *EGFR* alterations coexist with *EGFR* wildtype (*EGFR*wt), with tumors showing not only highly variable *EGFR* copy numbers, but also variable variant allele frequency of ECD missense mutations and *EGFRvIII* expression level. Epidermal growth factor receptor amplification occurs at a much lower incidence in LUAD (~5%).^10^ Additionally, the *EGFR* mutations responsive to *EGFR* inhibitors are typically found in the intracellular tyrosine kinase domain.^11^ These *EGFR* mutations in LUAD result in a constitutively active form of the receptor protein which, in contrast to ECD missense mutations in GBM, are ligand-independent.

To examine pathway differences between GBM and LUAD, we compared co-regulated EGFR genes between these tumor types. We specifically investigated the dynamics of *EGFR* expression levels in relation to common mutations therein (*EGFRvIII* and the ligand-sensitive mutations). Using multiple large-scale datasets, our work helps understand the molecular mechanisms of EGFR activation that are specific to GBM, which might benefit clinical practice.

## Materials and Methods

### Data Collection

For both IDH-wildtype (IDHwt) GBM and LUAD, The Cancer Genome Atlas (TCGA) read counts profiled using poly-A+ enriched RNA-sequencing (RNA-seq) were obtained from the Genomic Data Commons (GDC) data portal.^12^*EGFR/IDH* mutation, amplification status calls, and a fraction of *EGFRvIII* counts for TCGA-GBM were collected from the respective TCGA publication.^13^*EGFR* mutation and amplification status for the TCGA-LUAD dataset were retrieved from cBioPortal (http://cbioportal.org/). Sequencing data collection, preprocessing, and single nucleotide variant (SNV)/copy number variant (CNV) calling of samples from the Intellance-2/EORTC_1410 (EGAS00001005437) phase II trial on *EGFR*-amplified recurrent GBM are described elsewhere.^4,5^

Single-cell and single-nucleus RNA-seq (sc/snRNA-seq) IDHwt GBM data (*n* = 34) were obtained from 3 publicly available datasets. The *Van Hijfte* (*n* = 1, EGAD00001009871) and peri-tumoral neuronal-rich *Bolleboom-Gao* (*n* = 1, EGAD00001009964) datasets were generated in-house.^14^ Additional sc/snRNA-seq datasets were obtained from *CPTAC-3* (*n* = 18) and the *Diaz/Wang* (*n* = 14) public repositories.^15,16^ tSNE values and relative metamodule scores for adult IDHwt GBM scRNA-seq samples (*n* = 21) from the Neftel study were obtained from the Single Cell Portal at the Broad Institute (https://singlecell.broadinstitute.org/single_cell).^17^

### EGFR Amplifications and Mutations

GBM samples of both bulk datasets were considered to harbor an *EGFR*-activating mutation in case of an ECD mutation in R108, A289, G598, or *EGFRvIII*.^5^ Using the splice-variant specific junction counts, tumors were classified as *EGFRvIII* expressing for read count ratios (*EGFRvIII*/(*EGFRvIII* + *EGFR*wt)) greater than 0.01.^18^ High-copy number amplification regions and associated enhancers were obtained elsewhere.^4,19^ In LUAD, in-frame deletions in exon 19 (E746_A750del/L747_E749del/L747_T751del), L858R and G719C have shown clinical response to tyrosine kinase inhibitors and were considered *EGFR*-activating mutations (https://www.mycancergenome.org/).

### Bulk RNA-seq Data Processing

Samples with a library size containing less than 750 000 counted reads were considered of insufficient resolution, and were therefore excluded. Low count genes with a mean count per sample of less than 3 were excluded. Only ENSEMBL protein-coding genes were used for all analyses. Median-of-ratios normalization implemented in the DESeq2 package (v1.36.0) was applied to normalize RNA-seq data.^20^ The vst function in the DESeq2 package was used for variance-stabilizing transformation.

### Differential Gene Expression Analysis

Differentially expressed genes were screened with the Wald test for hypothesis testing as implemented in the DESeq2 package (v1.36.0).^20^ Contribution of variables to the fitted model was assessed with the coef function from the DESeq2 package. For each gene, the log_2_ fold change divided by its standard error was used to compare the outcome of different DESeq2 tests between LUAD and GBM. g:Profiler was used for pathway analysis of differentially expressed genes (https://biit.cs.ut.ee/gprofiler/).

### Sc/snRNA-seq Analysis

Normalization, preprocessing, and cell cluster identification were performed as described earlier.^14^ Cell types were assigned to their corresponding cell clusters based on expression values of established marker genes.^21,22^ Only samples with sufficient neuronal marker expression (*n* > 25 cells), cell types, and of high depth were included in further analyses (*van Hijfte n* = 1, *Bolleboom-Gao n* = 1, *Diaz/Wang n* = 1, CPTAC-3 *n* = 7).

### Random Forest Model

Random forest regression models for predicting *EGFR* expression levels were built using the ranger method as implemented in the caret R package.^23^ Prior to training regression models, gene selection steps were performed to reduce model complexity. The workflow for gene selection using correlation filtering, median absolute deviation, and the Boruta algorithm is detailed in Supplementary Methods. Genes identified by the Boruta algorithm as putative (no convergence after 100 runs) and confirmed (*P* < .01) important were considered predictive for *EGFR* expression (significant EGFR predictor [SEP] genes). To increase the robustness of our findings, results were averaged over 90 models. Our complete modeling workflow is described in Supplementary Methods.

### Cell Culture

We tested 2 patient-derived IDH-wildtype GBM cell lines, GS-216 and GS-1191.^24,25^ The use of patient tissue was approved by the Medical Ethical Review Committee Erasmus MC, code MEC-2013-090, and all patients provided informed consent in accordance with institutional guidelines. The cell culture procedures were adopted from a published protocol.^26,27^ Cells were cultured in Dulbecco’s modified Eagle medium/Nutrient Mixture F-12 (DMEM/F-12, 11320-033, Gibco, USA). Unless stated otherwise, the experiment utilized 20 ng/mL of basic fibroblast growth factor, 20 ng/mL of EGF, and 5 µg/mL of heparin in the medium. Extracellular matrix (ECM) coating (Cultrex Reduced Growth Factor Basement Membrane Extract, PathClear, R&D Systems) solution (1:100, diluted in culture media) was used to cover the surface of flasks.

### Internalization Experiments and Image Analysis

To study the internalization of EGFR, cells were first seeded in 96-well plates. After the cells were attached, the medium was taken off and replaced by a medium without ligands (starve medium, 24 hours). Afterward, cells were stimulated (200 ng/mL, diluted in culture media) with EGF (GibcoTM Human EGF Recombinant Protein, PHG0311, Fisher Scientific), BTC (Betacellulin human, B3670, Sigma-Aldrich), EREG (Recombinant Human Epiregulin Protein, 1195-EP-025/CF, R&D Systems), or PBS as negative control. Cells were fixed with paraformaldehyde (PFA) solution (4%, buffered, pH 6.9, 1.00496.5000, Sigma-Aldrich) at different time points after stimulation (0 minutes, 15 minutes, and 2 hours). To visualize EGFR presence and localization, fixed cells were stained with anti-EGFR (M3563, DAKO, 1:400 dilution) and Alexa Fluor 647 (A21240, Invitrogen, 1:500 dilution). Nuclei were stained with Hoechst 33342 (1:5000 dilution). Images were acquired using an Opera Phenix high-throughput high-content confocal microscope (PerkinElmer). At least 10 images were obtained per well, which maximized accurate quantification. Image analysis was conducted with Harmony software (PerkinElmer), applying uniform settings for all conditions in each experiment. Using the software, we used a multistep algorithm to quantify the number of intracellular EGFR spots. These spots were detected by finding the nucleus of the cell. Included parameters were nearest object distance, spot area, spot roundness, and spot intensity value.

### Data Processing and Visualization

Data processing and visualization were performed using the R programming language within Rstudio (v4.2.1) and the tidyverse R package (v1.3.2).^28^ The EGFR locus was plotted using the gggenes R package. Correlation plots were made with the recursive correlation-based clustering method (https://github.com/yhoogstrate/recursiveCorPlot).^14^

## Results

### Sample Overview

An overview of the *EGFR* mutational burden in the 3 datasets: IDHwt TCGA-GBM (*n* = 142), Intellance-2 (*n* = 211), and TCGA-LUAD (*n* = 508) is presented in Table 1. Sample selection steps for the TCGA-GBM and TCGA-LUAD datasets are described in Supplementary Figure 1.

**Table 1.** Incidence of *EGFR* Amplification and Activating Mutations Across the TCGA-GBM, Intellance-2, and TCGA-LUAD Datasets

		EGFR-Activating SNV	EGFRvIII	BTC Expression	EREG Expression	*n*
TCGA (GBM)	EGFR amplified	Absent	Negative	4.76	4.78	28
		R108/A289/G598	Negative	5.33	4.82	18
		Absent	Positive	4.82	4.82	18
		R108/A289/G598	Positive	5.23	4.74	7
	EGFR non-amplified	Absent	Negative	6.42	5.14	64
		R108/A289/G598	Negative	7.94	4.94	5
		Absent	Positive	8.39	5.40	2
Intellance-2 (GBM)	EGFR amplified	Absent	Negative	3.81	3.90	76
		R108/A289/G598	Negative	3.77	3.95	27
		Absent	Positive	3.79	3.93	68
		R108/A289/G598	Positive	3.44	3.96	17
	EGFR non-amplified	Absent	Negative	5.15	4.67	22
		R108/A289/G598	Negative	4.80	6.11	1
TCGA (LUAD)	EGFR amplified	Absent		8.74	8.13	16
		ex19_del/L858R/G719S/A/C		8.48	8.46	10
	EGFR non-amplified	Absent		8.82	7.24	451
		ex19_del/L858R/G719S/A/C		8.71	6.70	31

Median expression levels of the ligands *BTC* and *EREG* are also indicated. Abbreviations: EGFR, epidermal growth factor receptor; GBM, glioblastoma; LUAD, lung adenocarcinoma; TCGA, The Cancer Genome Atlas; VST, variance-stabilizing transformation.

*EGFR* amplifications were detected in 50% of the TCGA-GBM and 89% of the Intellance-2 samples.^29^ This difference fits with the patient selection criterion from these cohorts since *EGFR* amplification was an inclusion criterium for the Intellance-2 phase II trial. The TCGA-GBM dataset was therefore better suited to analyze molecular differences between *EGFR* amplification and *EGFR*wt, while the Intellance-2 dataset was suitable for associations with the level of *EGFR* amplification.

Within the *EGFR*-amplified TCGA-GBM samples, 35% carried the ligand-independent *EGFRvIII* mutation. The A289 (18%) and G598 (15%) activating missense mutations occurred at a similar but higher frequency compared with the R108 mutation (6%). *EGFRvIII* mutations were detected in 45% of the *EGFR*-amplified Intellance-2 samples. Also in this dataset, A289 was the most abundant activating missense mutation (14%) compared with G598 (7%) and R108 (3%).^5^

*EGFR* amplification was present in 5% of the TCGA-LUAD samples. In line with our expectations, the TCGA-LUAD dataset contained a high number of samples without *EGFR*-activating alterations (89%) compared with the TCGA-GBM dataset (46%). Of all *EGFR* mutations in LUAD samples, the L858R mutation was the most abundant (51%) compared with in-frame deletions in exon 19 (41%) and G719S/A/C (7%). The proportion of TCGA-LUAD tumors with an *EGFR*-activating mutation was significantly higher in the *EGFR*-amplified group (38%) compared with the *EGFR* non-amplified group (6%) (*P* = 8.05e−06, Fisher’s exact test). The co-occurrence of *EGFR* amplification and exon 19 deletions has been described earlier.^30^

### Random Forest Reveals Genes Associated With *EGFR* Expression

**Figure 1.**
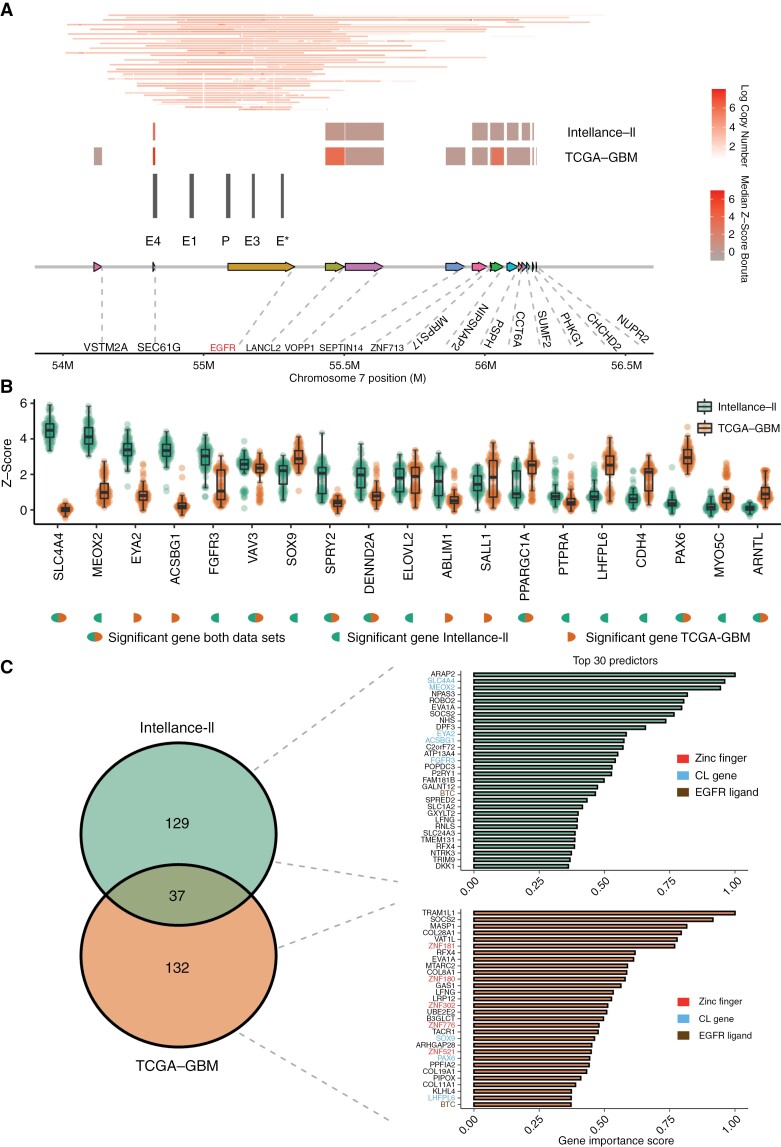
(A) (From bottom to top) Overview of the *EGFR* locus with common amplifications and putative enhancer locations. Median Boruta *Z*-scores from our predictive modeling approach on the TCGA-GBM and Intellance-2 datasets, with *EGFR* neighbors still included in the respective models. Amplification is skewed toward *SEC61G*, a gene highly contributing to the predictive models. (B) Median Boruta *Z*-scores of classical subtype genes (*N* = 19) from co-amplification revised models on the TCGA-GBM and Intellance-2 datasets. (C) Venn diagram indicating the number of SEP-genes on the Intellance-2 (*N* = 166) and TCGA-GBM (*N* = 169) datasets (37 overlapping). Gene importance scores for the top 30 predictors are depicted. Zinc finger genes, classical subtype genes, and EGFR ligands are highlighted. Abbreviations: EGFR, epidermal growth factor receptor; SEP, significant EGFR predictor.

We first aimed to find genes co-regulated with *EGFR* and performed random forest regression modeling to predict *EGFR* expression. Models were devised on each dataset separately. The purpose was to elucidate which genes mainly contributed to the prediction of *EGFR* expression and therefore might exhibit a strong association with *EGFR*. In our unadjusted models devised on the TCGA-GBM dataset, the strongest contributing genes (*R*^2^ = 0.73) were those neighboring *EGFR*, including *SEC61G* and *LANCL2* (Supplementary Figure 2A). *SEC61G*, but not *LANCL2*, was also considered of importance in models devised on the Intellance-2 dataset (*R*^2^ = 0.66, Supplementary Figure 2A). Neighboring genes of *EGFR* are often co-amplified on the ecDNA fragment. These genes may not contribute functionally to the *EGFR* network as they may be bystanders of the co-amplification (Figure 1A). While identification of these genes confirms the validity of our approach, they diminish insight into the effect of co-expressed genes by other regulatory mechanisms. Neighboring genes of *EGFR* (chr7p11.2) were therefore excluded from our analysis (Supplementary Data).

The co-amplification revised model reported 169 and 166 SEP genes as important in predicting *EGFR* expression, respectively, on the TCGA-GBM and Intellance-2 datasets (Figure 1B and C, Supplementary Data). The co-amplification revised performance was 0.67 on the TCGA-GBM dataset and 0.66 on the Intellance-2 dataset. 37 SEP-genes were overlapping between both models (bootstrapped *P*-value <.0001, Monte Carlo simulation). Since we noted considerable overlap in SEP-genes across the models, we wanted to elucidate the level of correlation of the signal by the nonoverlapping genes. To achieve this, we selected SEP-genes specific per dataset and performed principal component analysis (PCA) on these in both datasets (Supplementary Figure 2B). Independent PCA on the 2 lists of SEP-genes revealed a high Spearman correlation (>0.80) between their respective first principal components. Despite models for each dataset considered different genes for prediction of *EGFR* levels, both SEP-gene sets showed similarity in terms of their expression pattern.

To get an impression of possible shared mechanisms behind the genes responsible for predicting *EGFR* expression levels, we performed pathway enrichment analysis. Significant EGFR predictor-genes obtained from the TCGA-GBM were mainly involved in transcription regulation, while enrichment for negative regulation of signal transduction was found on SEP-genes from the Intellance-2 dataset.

Transcriptional GBM subtype clustering on bulk RNA-seq data is based on a 50-gene signature and distinguishes 3 classes, namely the classical, mesenchymal, and proneural GBM subtypes.^31^ Eleven (11/169) and fifteen (15/166) classical subtype genes were among the SEP-genes, respectively, on the TCGA-GBM and Intellance-2 datasets. The list of 37 overlapping SEP-genes contained a high proportion (*n* = 7) of classical subtype genes (bootstrapped *P*-value = .01, Monte Carlo simulation) including *ABLIM1*, *ELOVL2*, *FGFR3*, *LHFPL6*, *PPARGC1A*, *SOX9*, and *VAV3*. This relatively large intersect is likely because classical subtype genes are associated with high-level amplification of *EGFR.*^32^ Classical subtype genes considered SEP-gene in either one of the datasets (*n* = 19) showed distinct associations with *EGFR* (Figure 1B).

Recent single-cell RNA-seq data have identified 4 distinct phenotypic states of GBM cells.^17^ Out of 169 SEP-genes obtained from models devised on the TCGA-GBM dataset, *EDNRB* was the only gene associated with the astrocyte (AC)-like state. On the Intellance-2 dataset, *HEPN1*, *HOPX*, *BCAN*, *AQP4*, and *NDRG2* were members of genes involved in the AC-like state. The AC-like state (*n* = 39 genes) is associated with strong upregulation of *EGFR* and largely encompasses genes expressed by AC.^17^ In conclusion, traditional bulk and single-cell-based signatures only partially explained putative associations of the SEP-genes.

**Figure 2.**
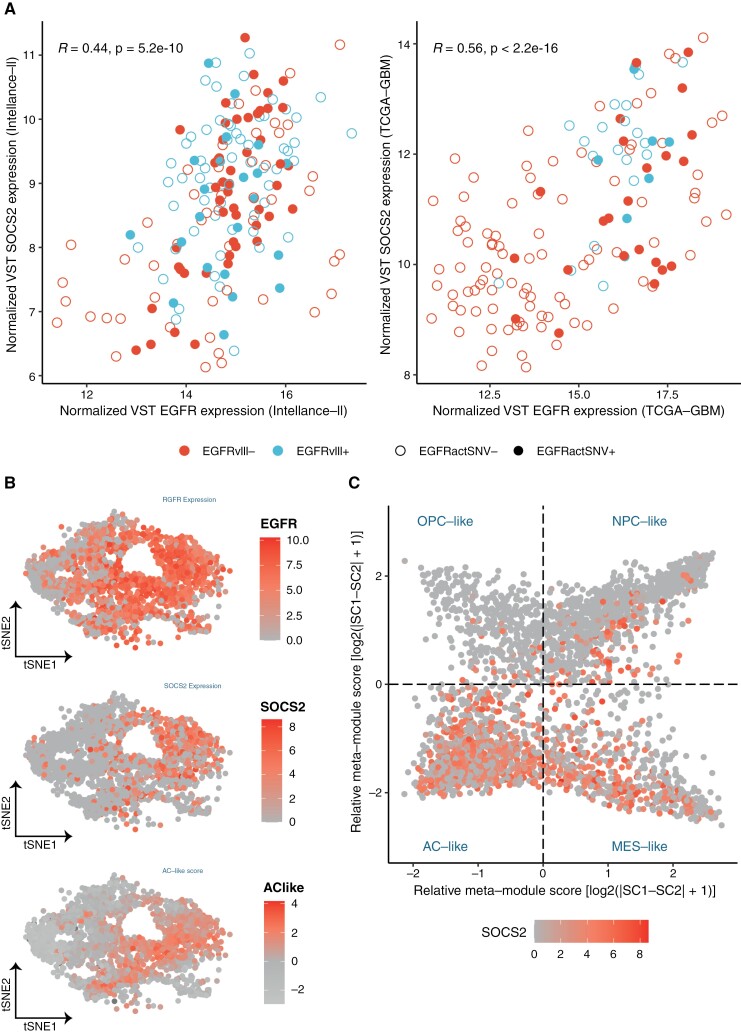
(A) Relation between normalized VST *EGFR* expression (*x*-axis) and *SOCS2* expression (*y*-axis) on the Intellance-2 (left) and TCGA-GBM (right) datasets. *EGFRvIII* and *EGFR*-activating missense mutations are indicated. (B) (From top to bottom) *EGFR* expression, *SOCS2* expression, and AC-like score on malignant cells derived from scRNA-seq adult GBM samples (*N* = 21) from Neftel et al. (C) Relative meta-module scores dividing cells in OPC-, NPC-, AC-, and MES-like states. Cells are indicated based on *SOCS2* expression levels. Abbreviations: AC, astrocyte; EGFR, epidermal growth factor receptor; GBM, glioblastoma; MES, mesenchymal; NPC, neural-progenitor; OPC, oligodendrocyte-progenitor; VST, variance-stabilizing transformation.

We observed that 1 gene in particular, namely Suppressor of Cytokine Signaling (SOCS2), was consistently identified as SEP-gene on both the Intellance-2 (90/90 models) and TCGA-GBM (88/90) datasets. *SOCS2* is positively correlated with *EGFR* (Figure 2A). *SOCS2* is reported to be a negative regulator of cytokine receptor signaling through the *JAK*/*STAT* pathway.^33^ It is therefore possible that such a negative feedback mechanism between *EGFR* and *SOCS2* exists.

Tumor cells enriched in the AC-like state showed a high expression of *SOCS2*, although *SOCS2* is not a member of the published AC-like signature genes (Figure 2B and C).^17^ Differential gene expression (DGE) analysis also showed a strong upregulation of *SOCS2* in *EGFR*-amplified TCGA-GBM samples (logarithmic fold change [LFC] = 1.99, false discovery rate [FDR]-adjusted *P*-value = 1.90e^−22^). To explore whether H3K27ac might play a role in regulating SOCS2 in the context of *EGFR* amplification, we interrogated H3K27 acetylation ChiP-seq data from GBM (Supplementary Methods). We found no significant differences in H3K827ac read counts across the investigated *SOCS2* regions between *EGFR*-amplified (*n* = 9) and *EGFR*wt (*n* = 32) patient-derived cell cultures (Supplementary Table 1 and Supplementary Figure 3).^34^

### EGFR Ligands BTC and EREG Inversely Correlate With EGFR Amplification

**Figure 3.**
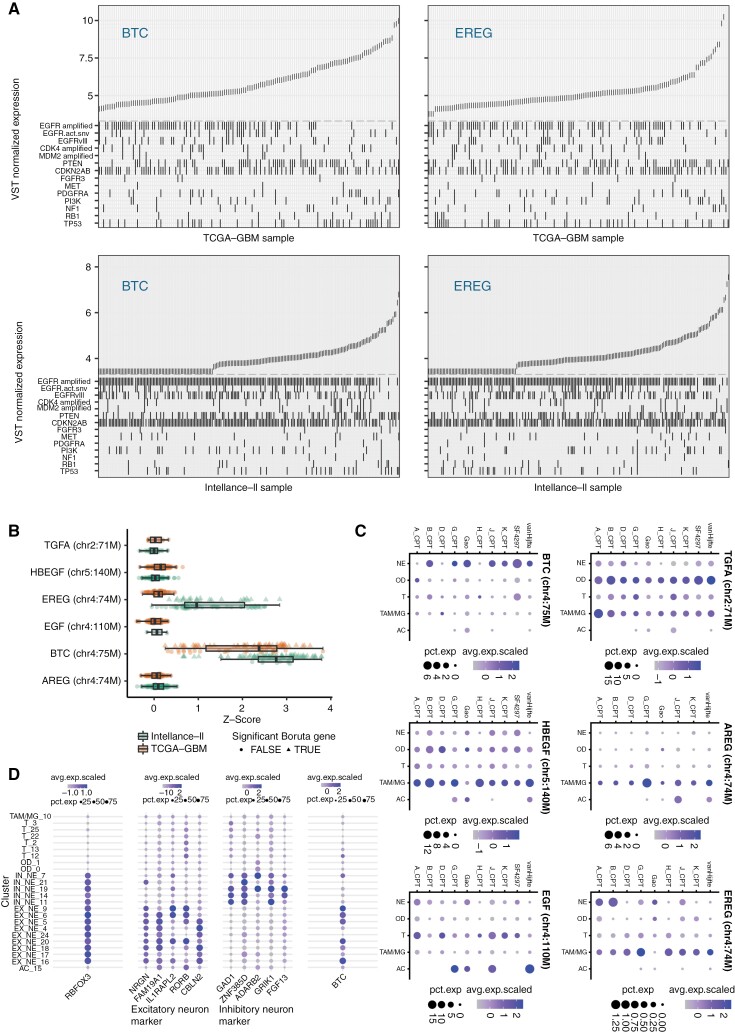
(A) Ordered VST normalized expression of *BTC* (left) and *EREG* (right) on the TCGA-GBM (top) and Intellance-2 datasets (bottom). Mutation statuses are indicated underneath. (B) Boruta *Z*-scores of EGFR ligands (*TGFA*, *HBEGF*, *EREG*, *EGF*, *BTC*, and *AREG*) from 90 models built on the Intellance-2 and TCGA-GBM. For each model, Boruta’s decision to consider genes’ contribution significant is indicated. (C) *EGFR* ligand expression in neurons (NE), oligodendrocytes (OD), tumor cells (T), (tumor-associated) macrophages/microglia (TAM/MG), and astrocytes (AC) across multiple sc/sn-RNA-seq datasets. (D) Expression levels of the neuron marker *RBFOX3*, inhibitory and excitatory neuron markers, and BTC in the Bolleboom-Gao snRNA-seq dataset. Abbreviations: EGFR, epidermal growth factor receptor; VST, variance-stabilizing transformation.

Interestingly, 2 EGFR ligands were identified as SEP-genes: *betacellulin* (*BTC*) and *epiregulin* (*EREG*) (Figure 3A and B). *BTC* was identified in both TCGA and Intellance-2 datasets whereas *EREG* was identified only in the Intellance-2 dataset (Figure 1). Samples with high *BTC* expression showed a significantly lower EGFR copy number (*P* = 8.33e^−9^, Wilcoxon test on median expression level TCGA-GBM) and EGFR-amplified samples showed significant downregulation of *BTC* in a DGE analysis in both the TCGA-GBM (LFC = −2.51, FDR-adjusted *P*-value = 6.74e−15) and Intellance-2 datasets (LFC = −3.07, FDR-adjusted *P*-value = 2.45e−08). *EREG* showed a negative and nonlinear relationship with *EGFR* where very high *EREG* expression (95th percentile) was almost exclusively found in samples with low *EGFR* expression (below the median expression) (Supplementary Figure 4). Although *EREG* was identified by random forest regression only in the Intellance-2 dataset, the gene was downregulated in EGFR-amplified samples in both the TCGA-GBM (LFC = −1.92, FDR-adjusted *P*-value = 1.24e−06) and Intellance-2 (LFC = −2.53, FDR-adjusted *P*-value = 1.16e−05) datasets using DGE. None of the other 4 *EGFR* ligands were differentially expressed (FDR-adjusted *P*-value <.01 and |LFC| >1.5) in either dataset (Supplementary Data).

In addition, we found that *EGFR-*activating mutations were less frequently observed at high *EREG* and *BTC* expression (Figure 3A). The inverse correlation of EGFR amplification levels with the expression of EGFR ligands and the inverse correlation of EGFR ligand expression with the presence of ligand-sensitizing mutations indicate tumors employ various modes to activate the receptor. These results imply that receptor activation remains important after tumor initiation.

We performed snRNA-seq and screened public-domain data to determine the source of expression of the different EGFR ligands. For each of the ligands, average expression values per cell type are visualized in Figure 3C. In agreement with Guo et al., we find that the expression of the EGFR ligands is low.^35^ We also find that the source of expression differed between different EGFR ligands and was not specific to tumor cells. Neurons were the primary source of *BTC* expression, with expression predominantly in excitatory neurons (Figure 3D). *EREG* was mainly expressed by (tumor-associated) macrophages/microglia.

**Figure 4.**
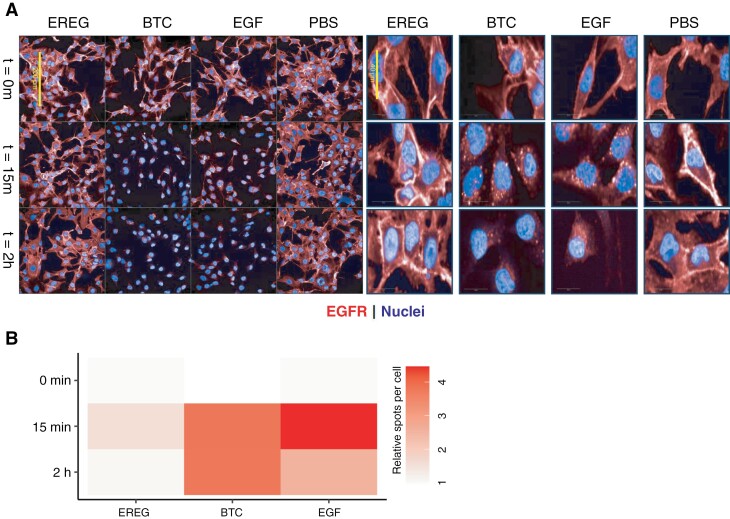
(A) Representative confocal microscopic images of EGFR at multiple time points (0 minutes, 15 minutes, and 2 hours) after stimulation with EREG, BTC, EGF, and PBS as negative control. The right panel displays zoomed-in images showing intracellular accumulation of EGFR (spots) after 15 minutes and 2 hours of ligand stimulation. (B) quantitative analysis of microscopic images by a multistep algorithm showing the PBS-normalized number of spots per nucleus for each condition. Results are averaged across 2 cell lines, with each experiment conducted in replicate. Abbreviation: EGFR, epidermal growth factor receptor.

To examine potential differences in EGFR internalization following activation by the various ligands, we cultured primary GBM (GS.1191 and GS.0216) and stimulated them with ligands EGF, EREG, or BTC. After ligand stimulation (15 minutes and 2 hours) we observed receptor internalization as seen by the appearance of intracellular EGFR protein ‘spots’ (Figure 4A). Internalization after ligand stimulation was also confirmed in earlier work using FACS, where a significant decrease in cell surface EGFR levels was observed upon ligand binding.^36^ We observed that the number of EGFR protein spots was markedly lower for EREG, which is consistent with its low binding affinity and resulting in weaker activation, as noted in our previous work.^5^ Despite the lower number of spots, the internalization route seemed similar across all ligands. We quantified the number of spots per nucleus and did not observe substantial differences in internalization patterns between ligands (Figure 4B). Furthermore, in our earlier work, we tested receptor activation across a broader spectrum of EGFR ligands in ECD mutation constructs. This included high-affinity ligands (EGF, TGFα, HB-EGF, and BTC) and low-affinity ligands (AREG, EREG, and EPGN). While EGFRwt showed strong activation only with high-affinity ligands, ECD-mutated variants exhibited enhanced sensitivity and strong activation even with low-affinity ligands, such as AREG, EREG, and EPGN.

### Key p53/rb1 Pathway Genes Are Downregulated in Samples With an *EGFR*-Activating SNV

**Figure 5.**
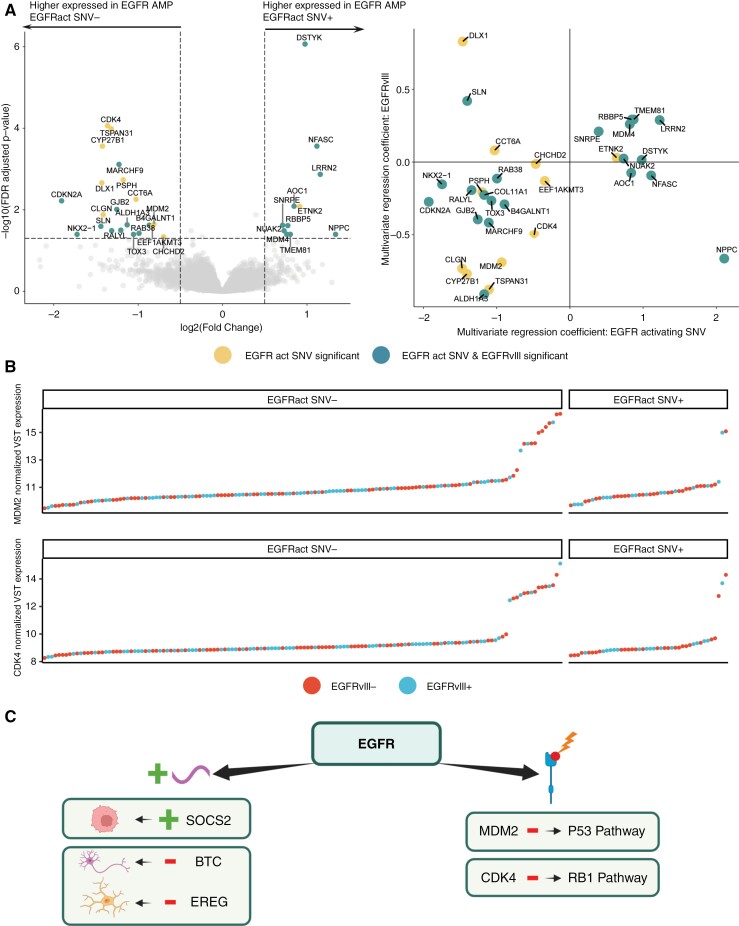
(A) Left: Volcano plot of the differential gene expression test between *EGFR*-amplified samples with or without *EGFR*-activating missense mutations. The *x*-axis indicates the log_2_ fold change of each gene and the *y*-axis the logarithmic FDR-adjusted *P*-value. Significant genes from this test and an external *EGFRvIII* study are indicated. Right: Regression coefficients of multivariate differential expression tests for *EGFR*-activating SNVs (*x*-axis) and *EGFRvIII* (*y*-axis). (B) Ordered VST normalized expression values for *MDM2* (top) and *CDK4* (bottom) in *EGFR*-amplified samples with or without an *EGFR*-activating SNV. Samples are colored by *EGFRvIII* status (positive: blue, negative: red). (C) Key findings on *EGFR* activation and its associated expression patterns as described in this study. Abbreviations: EGFR, epidermal growth factor receptor; VST, variance-stabilizing transformation.

Since activating *EGFR*-activating point mutations results in a receptor that is more sensitive to ligand stimulation,^5^ we further explored the effect of these mutations. For this, we performed DGE analysis on *EGFR*-amplified tumors from the Intellance-2 dataset to find *EGFR*-activating mutation-specific differential expression. In total, 32 genes were differentially expressed (|LFC| >0.5, FDR-adjusted *P*-value <.05) between *EGFR*-amplified tumors with and without an *EGFR*-activating point mutation (Figure 5A, Supplementary Data). Key negative regulators of the p53 (*CDKN2A* and *MDM2*) and RB1 (*CDKN2A* and *CDK4*) signaling pathways were significantly downregulated. There were fewer samples with very high *CDK4*/*MDM2* expression values in samples with an EGFR-activating SNV (Figure 5A). Interestingly, many (11/32) of these differentially expressed genes were also found in earlier work investigating the specific transcriptomic profile of *EGFRvIII*-mutated GBMs (*n* = 741).^4^ The multivariate regression coefficients for both *EGFRvIII* and EGFR-activating SNVs on the 32 identified genes indicated that both types of activating mutations contributed to the downregulation of *CDK4* and *MDM2*, as evidenced by their negative regression coefficients (Figure 5B). We thus observed similarity in terms of the associated expression patterns between *EGFRvIII* and activating SNVs, suggesting homologous functionality. This is further summarized in Figure 5C, which encapsulates our key findings on *EGFR* activation and its associated expression patterns from this study.

### High EGFR Ligand Expression Is Associated With Response to Depatux-m + TMZ

In our previous work, we identified that the presence of ligand-sensitive *EGFR* SNVs was associated with a favorable response to the combination of depatux-m and temozolomide (TMZ).^5^ Building upon these and our current findings, we investigated whether overall ligand expression correlates with treatment response. Our univariate analysis revealed that *EGFR*-amplified tumors with high overall ligand expression (above the median) showed a better response to depatux-m combined with TMZ compared with the control arm receiving TMZ or lomustine (CCNU) (hazard ratio [HR]: 0.53; 95% CI [0.31-0.91], *P* = .021). This was not observed in the group with low ligand expression (HR: 0.91; 95% CI [0.53-1.55], *P* = .73). The association between ligand expression and treatment response remained significant after adjusting for the known prognostic factors age and O6-methylguanine-methyltransferase (MGMT) methylation status (HR: 0.51; 95% CI [0.30-0.87], *P* = .015).

### *EGFR*-Activating Alterations Exhibit a Distinct Pattern Between GBM and LUAD

Since *EGFR*-activating mutations are also prevalent in LUAD, we performed similar analyses between mutation-positive and -negative samples in this tumor type (Supplementary Figure 5). Interestingly, our DGE analysis showed little overlap of identified genes between LUAD and GBM. Correlation between the outcome of their test statistics, that is the effect on the transcriptome of activating *EGFR* point mutations in GBM and LUAD, was surprisingly low (*R* = 0.081). Genes co-amplified with EGFR and its ligands showed concordant test statistics (Supplementary Figure 5). Conversely, whereas *SOCS2* was strongly associated with activating mutations in GBM (LFC = 1.91, FDR-adjusted *P*-value = 1.94e−15), it showed an inverse association to mutations in LUAD (LFC = −0.68, FDR-adjusted *P*-value = .007). This finding suggests a tissue-type-dependent association between *SOCS2* and *EGFR*. In conclusion, our results indicate that *EGFR*-activating alterations are associated with distinct *EGFR*-associated transcriptional programs between GBM and LUAD.

## Discussion

EGFR is an interesting potential treatment target in GBM due to the high incidence of *EGFR* alterations and the success of EGFR-targeted therapy in LUAD, where inhibitors are effective for specific types of mutations. This work aimed to deepen the understanding of EGFR activation and regulation mechanisms in GBM by revealing the complexity of its activation pathways, highlighting the challenges in targeting EGFR in GBM, particularly when compared with its more successful inhibition in LUAD. We modeled the co-regulation patterns of EGFR and examined their association with amplifications and activating mutations.

*EGFR-*activating mutations in LUAD lead to a ligand-independent constitutively active receptor. In GBM, the primary event is high-copy receptor amplification followed by either gain of missense mutations that result in ligand-hypersensitivity or deletions of exons 2-7 resulting in a ligand-independent constitutively active mutation. Both mutation types suggest a requirement for EGFR activation in GBMs. Moreover, ligand-independent constitutive signaling may also arise from high levels of EGFR amplification. In such cases, *EGFR* overexpression can lead to constitutive autophosphorylation without the need for ligand binding. The high abundance of EGFR receptors might hereby promote spontaneous receptor dimerization and activation.^37^ Besides ligand-independence and ligand-hypersensitive mechanisms, our results point toward a third, ligand-driven mechanism for EGFR activation through ligand expression. Out of more than 20 000 genes, our modeling workflow captured a negative association between *EGFR* and its ligands *BTC* and *EREG*. This finding was further confirmed by our DGE analysis. Our sc/sn-RNA-seq analyses indicated nonmalignant cells as the primary source of expression for these ligands. The negative association suggests a complex interplay between *EGFR*-expressing tumor cells and ligand-expressing stromal cells.

Earlier work noted similar *EGFR*-ligand levels for *EGFR*-amplified and non-*EGFR*-amplified GBMs.^35^ The clinical relevance of *EGFR* ligands was underlined since high ligand expression was associated with an improved prognosis in *EGFR*-amplified GBM.^35^ The same work also discovered that an increased ligand availability resulted in smaller tumors, decreased invasion, and improved survival in mice. Additionally, we and others have demonstrated that ECD mutations enhance receptor sensitivity to low-affinity ligands, leading to a stronger activation compared with *EGFRwt*.^5,38^ This enhanced activation seems not solely driven by increased ligand binding of low-affinity ligands in *EGFR*-mutated variants.^39^ Our retrospective analysis of data from the Intellance-2 trial, along with our previous study,^5^ demonstrates that both high *EGFR* ligand expression and *EGFR* SNVs are significantly associated with improved patient responses to depatux-m combined with TMZ in *EGFR*-amplified recurrent GBM. These findings indicate the preserved significance of ligands despite high expression levels of *EGFR.* Nevertheless, prospective studies are needed to evaluate the prognostic value of EGFR ligand expression in GBM.

*EGFR* activation in GBM seems to be modulated by the highly varying level of *EGFR* amplification, the sensitivity of the receptor toward the different ligands, and ligand expression levels. These factors likely determine whether EGFR oscillates between ligand-dependent and -independent models of activation, further increasing the complexity of the signaling dynamics in GBM. We hypothesized that activating mutations may result in a unique expression profile, as activating *EGFR* missense mutations show a distinct ligand-receptor binding affinity, have tumorigenic potential, and specific mutations are associated with survival.^38–40^ We found that oncogenes *CDK4* and *MDM2* involved in the p53/Rb pathways were specifically downregulated in the presence of *EGFR*-activating SNVs. A similar effect was observed in the presence of *EGFRvIII.*^4^ Neftel et al. suggested that specific genetic alterations lead to a favored state where amplification of *EGFR* leads to the AC-like state as the dominant state, and AC-like cells proliferated more upon overexpression of *EGFR* than *CDK4*. Activating mutations in *EGFR*, typically characterized as late events, might lead to a tumor that is more associated with the AC-like state leading to redundancy in high *CDK4* expression levels. However, since our analysis focused only on the most common A289, R108, and G598 mutations, it remains uncertain whether these findings extend to other ECD mutations, such as those in domain IV.^41^ Further studies are needed to explore their impact as the incidence in this cohort was too low to draw meaningful conclusions.

Although this study does not fully explain the differential response to EGFR inhibitors between LUAD and GBM, it provides valuable insights into the complexity of EGFR activation in GBM. In LUAD, EGFR inhibitors target constitutively active mutations, which are responsive to these treatments. In contrast, GBM exhibits a distinct EGFR activation landscape, also demonstrated by the different *EGFR*-associated signaling pathways. *EGFR*-activating alterations between both tumor types differ in their ligand dependence, meaning that the effect of EGFR ligands in GBM should receive more attention in follow-up studies. Our findings provide new insights into EGFR-associated transcriptional programs in GBM and suggest that a better understanding of the interplay between EGFR, its ligands, and other signaling pathways could be used for patient stratification and personalized treatment approaches.

## Supplementary Material

vdae229_suppl_Supplementary_Figure

vdae229_suppl_Supplementary_Materials

vdae229_suppl_Supplementary_Table

## Data Availability

Raw sequencing data obtained from the Intellance-2 study have been made available through the European Genome-phenome Archive (accession number: EGAS00001005437).
