# Supplementary material for: Transcriptomic analysis of EGFR co-expression and activation in glioblastoma reveals associations with its ligands
Source: Neurooncol Adv. 2024 Dec 28;7(1):vdae229. doi: 10.1093/noajnl/vdae229 (PMC11829203; doi:10.1093/noajnl/vdae229)
Supplement: vdae229_suppl_Supplementary_Figure [file vdae229_suppl_supplementary_figure.pptx]

## Slide 1
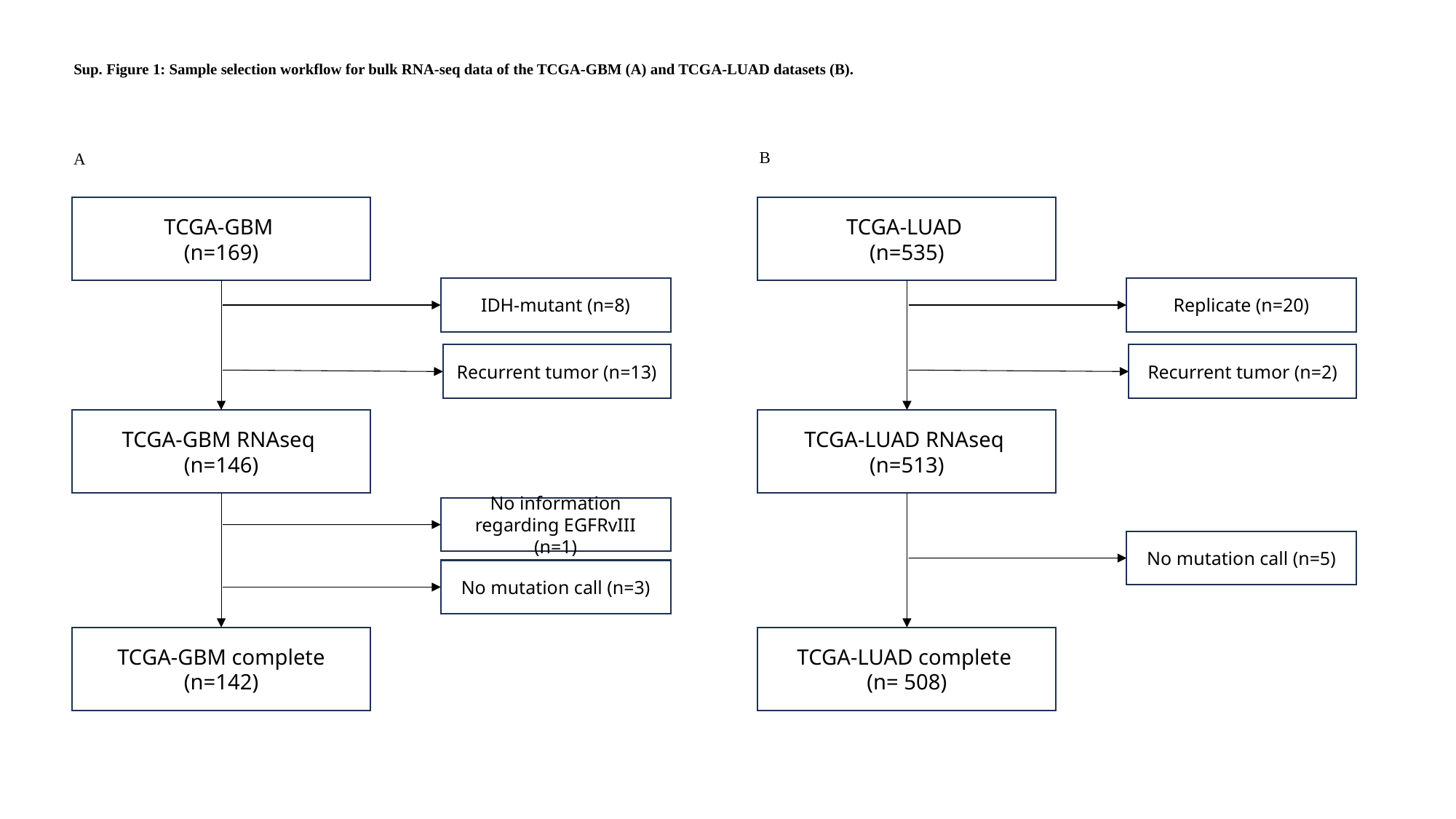

Sup. Figure 1: Sample selection workflow for bulk RNA-seq data of the TCGA-GBM (A) and TCGA-LUAD datasets (B).
B
A
TCGA-GBM
(n=169)
TCGA-LUAD
(n=535)
IDH-mutant (n=8)
Replicate (n=20)
Recurrent tumor (n=13)
Recurrent tumor (n=2)
TCGA-GBM RNAseq
(n=146)
TCGA-LUAD RNAseq
(n=513)
No information regarding EGFRvIII (n=1)
No mutation call (n=5)
No mutation call (n=3)
TCGA-GBM complete (n=142)
TCGA-LUAD complete
(n= 508)

## Slide 2
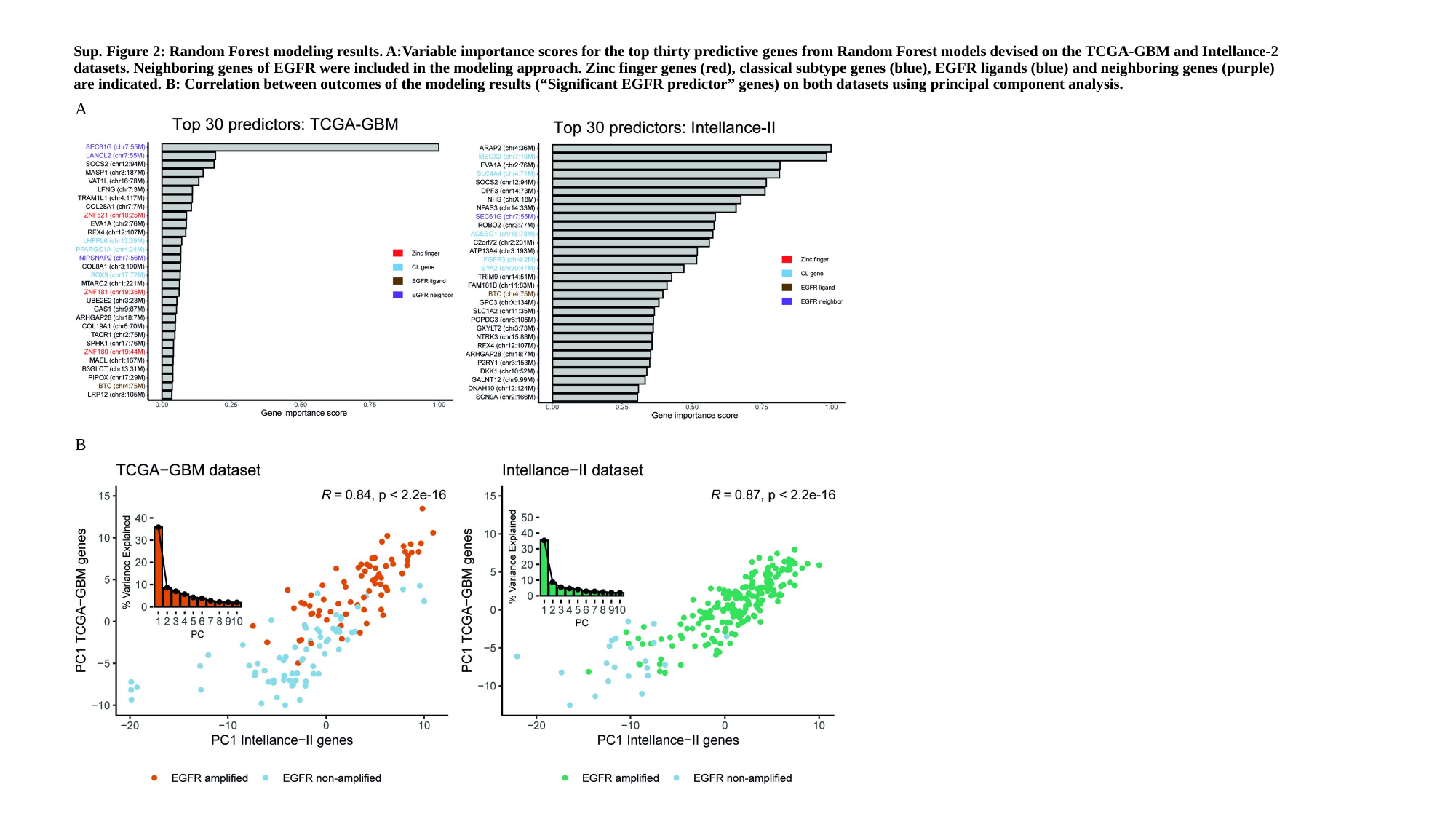

Sup. Figure 2: Random Forest modeling results. A:Variable importance scores for the top thirty predictive genes from Random Forest models devised on the TCGA-GBM and Intellance-2 datasets. Neighboring genes of EGFR were included in the modeling approach. Zinc finger genes (red), classical subtype genes (blue), EGFR ligands (blue) and neighboring genes (purple) are indicated. B: Correlation between outcomes of the modeling results (“Significant EGFR predictor” genes) on both datasets using principal component analysis.
A
B

## Slide 3
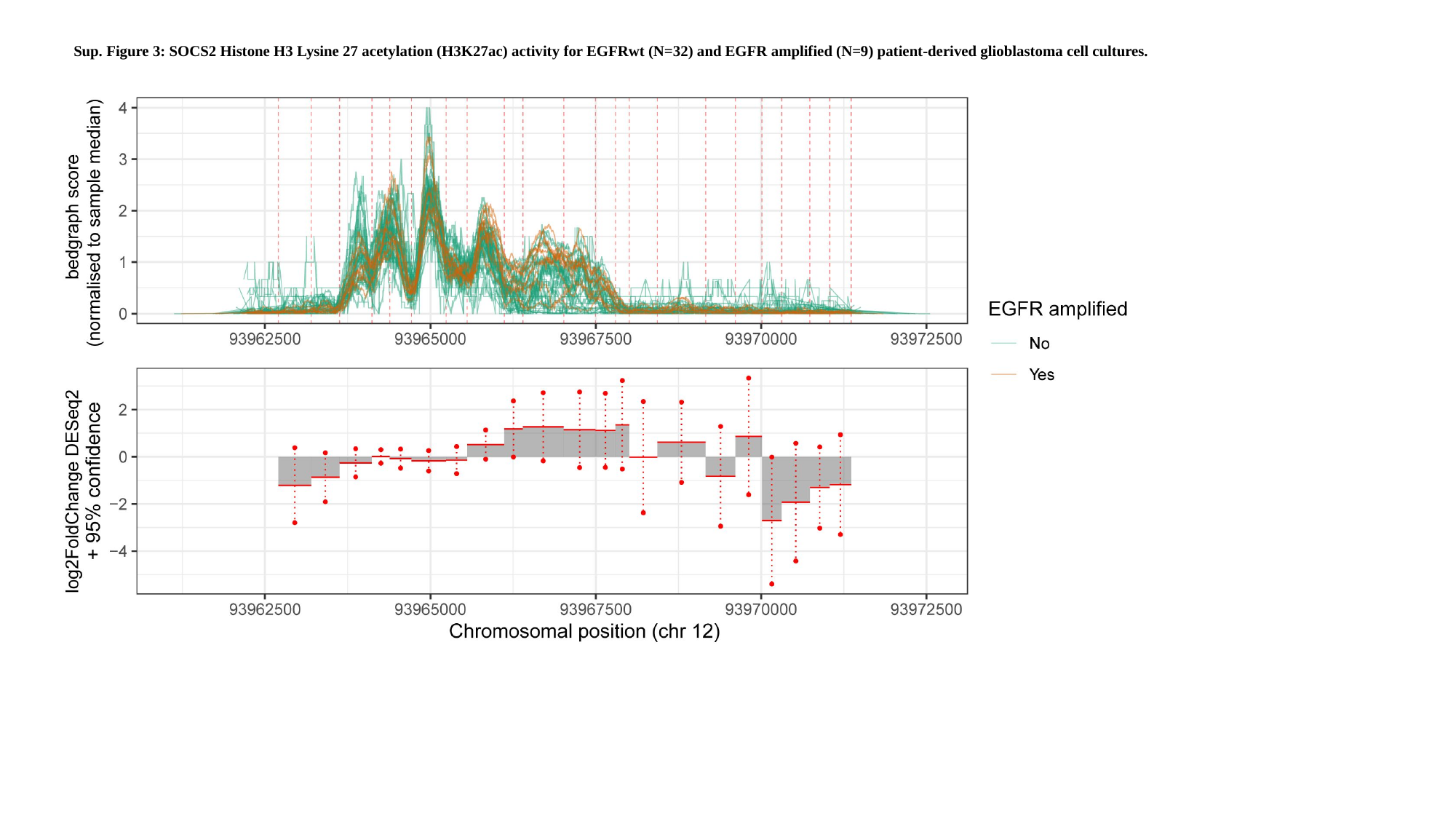

Sup. Figure 3: SOCS2 Histone H3 Lysine 27 acetylation (H3K27ac) activity for EGFRwt (N=32) and EGFR amplified (N=9) patient-derived glioblastoma cell cultures.

## Slide 4
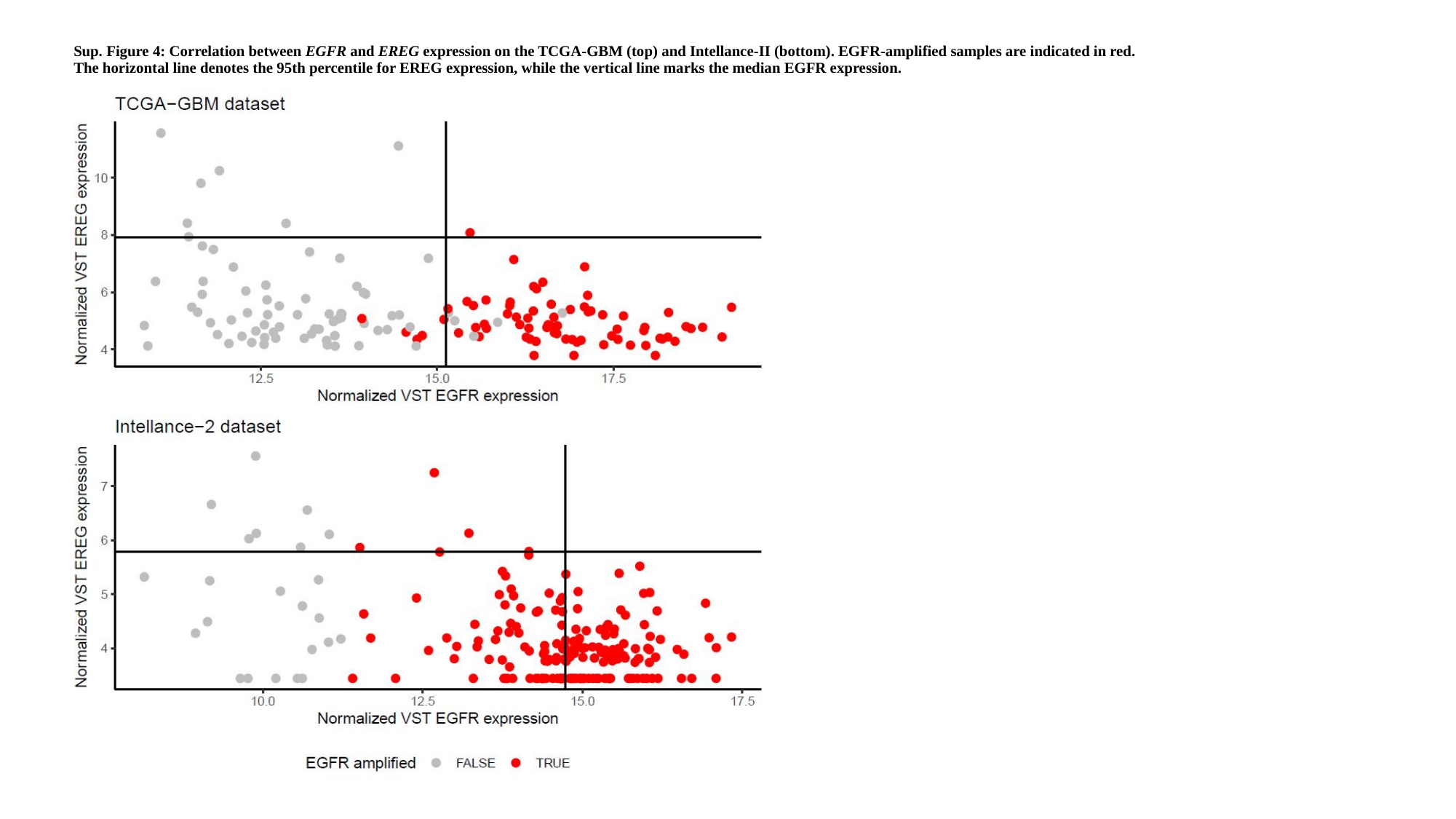

Sup. Figure 4: Correlation between EGFR and EREG expression on the TCGA-GBM (top) and Intellance-II (bottom). EGFR-amplified samples are indicated in red. The horizontal line denotes the 95th percentile for EREG expression, while the vertical line marks the median EGFR expression.

## Slide 5
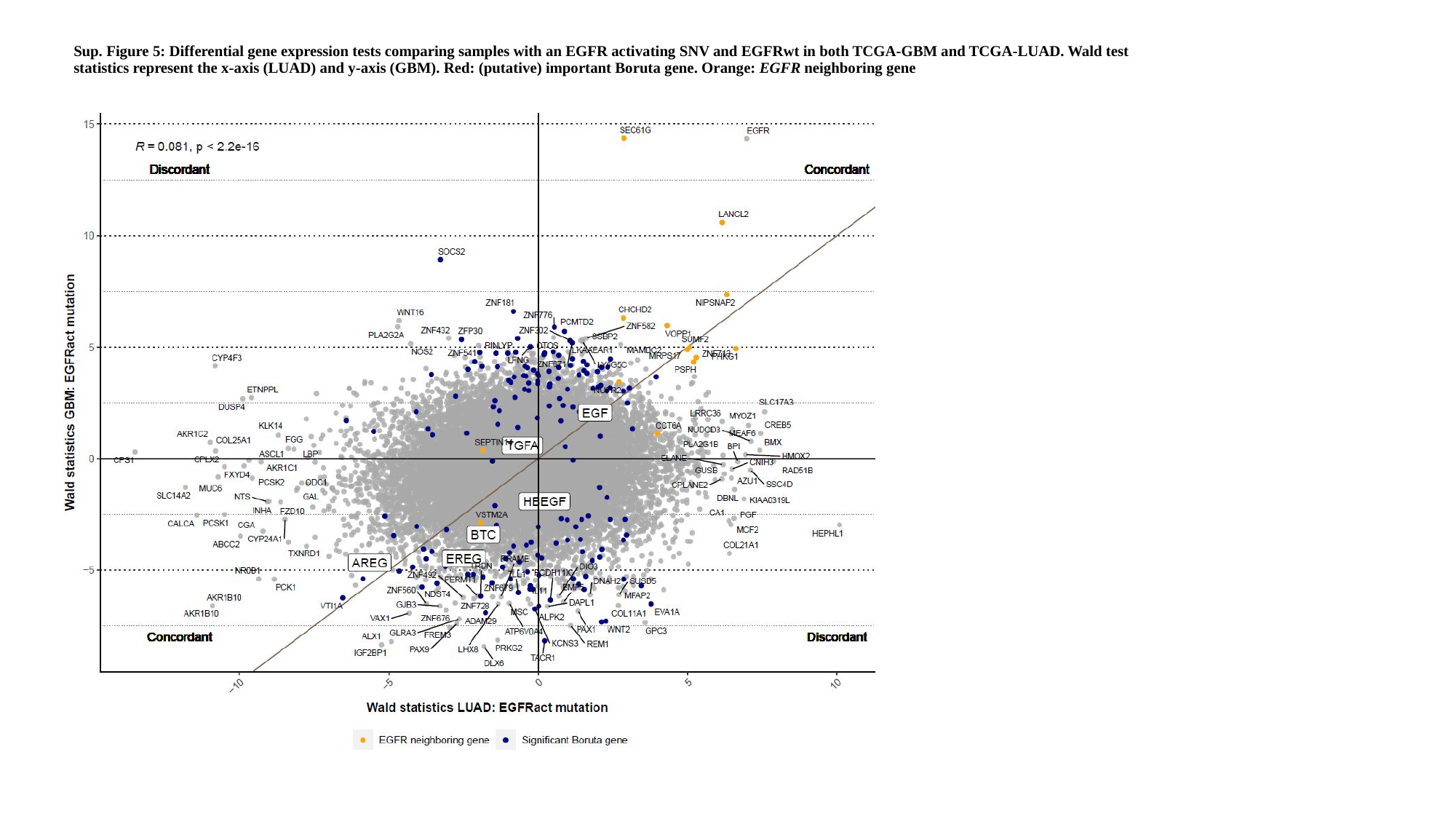

Sup. Figure 5: Differential gene expression tests comparing samples with an EGFR activating SNV and EGFRwt in both TCGA-GBM and TCGA-LUAD. Wald test statistics represent the x-axis (LUAD) and y-axis (GBM). Red: (putative) important Boruta gene. Orange: EGFR neighboring gene
