# Supplementary material for: Transcriptomic analysis of EGFR co-expression and activation in glioblastoma reveals associations with its ligands
Source: Neurooncol Adv. 2024 Dec 28;7(1):vdae229. doi: 10.1093/noajnl/vdae229 (PMC11829203; doi:10.1093/noajnl/vdae229)
Supplement: vdae229_suppl_Supplementary_Materials [file vdae229_suppl_supplementary_materials.docx]

# Supplementary Methods

## **Random Forest regression**

Cross-validation

Models were built independently on the TCGA-GBM and Intellance-II RNA-seq data sets. To fully make use of the data set an outer and inner cross-validation were combined. The outer cross validation loop was repeated (N=3), where for each fold the normalized and VST transformed data set was split into a train (85%) and test (15%) set, as implemented in the createDataPartition function from the caret package. The overall expression pattern of *EGFR* was preserved on both sets by creating balanced partitions. In the inner cross-validation loop, repeated (N=3) K-fold cross-validation (K=10) was applied on each of the train sets to obtain a stable list of genes. K-fold cross-validation means that models were iteratively trained on 90% of the train set and validated on the remaining 10%. Balanced cross-validation partitions were made using the CreateFolds function as implemented in the caret package. In summary, for each of the data sets, 90 models were evaluated to obtain a reliable list of predictive genes.

Parameters model training

For each model, the ranger method as implemented in the caret package was used for training. The minimum size of the terminal nodes was held constant at a value of 5 in order to control depth of the tree (min.node.size=5). Gene importance was assessed by the permutation score (importance=“permutation”). The optimal model was selected by minimizing the Root Mean Squared Error (RMSE).

Gene selection

Bulk RNA sequencing data are characterized by a large number of measured genes compared to the number of samples. In order to reduce overfitting caused by the high dimensionality, informative genes were selected before training the model. Genes with a very low correlation with *EGFR* were filtered (Spearman correlation R<0.10). Genes yielding low variability across the cohort (e.g. “housekeeping genes”) were considered low/non-informative. Therefore, genes with a median absolute deviation (MAD) < 0.25 were filtered. Lastly, the Random Forest based Boruta algorithm was used to find relevant genes.^1^ The Boruta analysis reported the importance of genes as either rejected (p>0.01), confirmed (p<0.01) or putative (no convergence after 100 runs). Boruta’s “putative” and “confirmed” were included for model training.

Contribution genes to model

The gene importance scores obtained from the 90 models as described above were combined to a single normalized weighted variable importance score (I) by using the mean importance score (S) and the number of times the gene was reported by the Boruta algorithm (Nboruta):

I = (S * Nboruta) / max(S)

**Copy number determination EGFR locus**

TCGA-GBM copy number calls were derived from the ICGC Data Portal release 25 (https://dcc.icgc.org/releases).^4^ Copy number values were calculated with the following formula:

$$copy number=2*2^{segment mean}$$

Copy number values were logarithmically (log2) transformed. We enriched for samples with high *EGFR* copy number amplification by filtering for samples with a segmented focal amplification call higher than 5. Analyses were performed using the human reference genome hg19.

**SOCS2 H3K27ac determination**

We interrogated publicly available H3K27ac ChIP-seq data from 41 samples derived from either glioblastoma primary tissue or stem cell models (GSE119755).^5^ Data was obtained as processed bedgraph files representing scaled regional intensities. Scaled intensities were converted to counts by dividing through the per-sample lowest intensity value larger than 0. The *SOCS2* region was segregated into 21 peak covering regions by visual inspection of the raw data. A per peak covering region count matrix was composed by including only regional intensities falling entirely within the peak covering bins. Counts and chromosomal regions are given (Supplementary Table 1). DESeq2 was used for differential analysis of the read counts, comparing samples harbouring *EGFR* amplifications with those lacking EGFR amplifications. P-values were multiple-testing corrected using FDR. For plotting, the intensities were normalised by dividing through the per sample median intensity. The DESeq2 Log2FoldChanges with their respective 95% confidence intervals were plotted.

**References**

1. Kursa MB, Rudnicki WR. *Feature Selection with the Boruta Package*. Vol 36.; 2010. Accessed November 11, 2020. http://www.jstatsoft.org/

2. Wang L, Babikir H, Müller S, et al. The phenotypes of proliferating glioblastoma cells reside on a single axis of variation. *Cancer Discov*. 2019

3. Couturier CP, Ayyadhury S, Le PU, et al. Single-cell RNA-seq reveals that glioblastoma recapitulates a normal neurodevelopmental hierarchy. *Nat Commun 2020 111*. 2020;11(1):1-19. doi:10.1038/s41467-020-17186-5

4. Brennan CW, Verhaak RGW, McKenna A, et al. The somatic genomic landscape of glioblastoma. *Cell*. 2013;155(2):462. doi:10.1016/j.cell.2013.09.034

5. Mack SC, Singh I, Wang X, et al. Chromatin landscapes reveal developmentally encoded transcriptional states that define human glioblastoma. *J Exp Med*. 2019;216(5):1071-1090. doi:10.1084/JEM.20190196

6. Hahne F, Ivanek R. Visualizing genomic data using Gviz and bioconductor. *Methods Mol Biol*. 2016;1418:335-351. doi:10.1007/978-1-4939-3578-9_16/
